# The Predictive Value of Computed Tomography and HA3D Nephrometry Scores for Complications After Partial Nephrectomy: A Prospective Pilot Study

**DOI:** 10.3390/cancers18071047

**Published:** 2026-03-24

**Authors:** Agostino Fraia, Sara Riolo, Francesco Di Bello, Salvatore Papi, Ivan Di Giulio, Giovanni Costa, Roberto Knez, Tommaso Silvestri, Bernardino de Concilio, Massimiliano Creta, Nicola Longo, Guglielmo Zeccolini, Antonio Celia

**Affiliations:** 1 Department of Neurosciences, Reproductive Sciences and Odontostomatology, University of Naples “Federico II”, 80131 Naples, Italy; salvatorepapi@blu.it (S.P.); massimiliano.creta@unina.it (M.C.); nicola.longo@unina.it (N.L.); 2 Department of Urology, La Sapienza University, 00185 Rome, Italy; sara.riolo@uniroma1.it; 3 Department of Urology, Fundació Puigvert, 08025 Barcelona, Spain; francesco.dibello@unina.it; 4 Department of Urology, San Bassiano Hospital, 36061 Bassano del Grappa, Italy; ivan.digiulio@aulss7.veneto.it (I.D.G.); giovanni.costa1@aulss7.veneto.it (G.C.); roberto.knez@aulss7.veneto.it (R.K.); tommaso.silvestri@aulss7.veneto.it (T.S.); b.deconcilio@aulss7.veneto.it (B.d.C.); guglielmo.zeccolini@aulss7.veneto.it (G.Z.); antonio.celia@aulss7.veneto.it (A.C.)

**Keywords:** partial nephrectomy, RENAL score, PADUA score, HA3D, nephrometry score, robotic surgery, laparoscopic surgery, renal tumor complexity, complications

## Abstract

**Background/Objectives**: Accurate preoperative assessment of renal tumor complexity is essential for surgical planning and for predicting perioperative outcomes after partial nephrectomy (PN). RENAL and PADUA nephrometry scores, traditionally derived from two-dimensional (2D) computed tomography (CT) imaging, are widely used to quantify renal tumor complexity and surgical risk. However, the introduction of hyperaccuracy three-dimensional (HA3D) models has enabled enhanced anatomical visualization, potentially improving the assessment of surgical difficulty and the prediction of postoperative complications. The aim of this study was to compare conventional CT-based RENAL and PADUA scores with HA3D-derived nephrometry scores in predicting perioperative complications in patients undergoing robot-assisted or laparoscopic PN. **Methods**: A total of 17 consecutive patients with intermediate- or high-complexity category renal tumors (RENAL ≥ 7) and moderate- or high-risk category tumors (PADUA ≥ 8) were prospectively enrolled. Preoperative demographic and clinical parameters, as well as intraoperative and postoperative data, were prospectively collected. Tumor characteristics were evaluated using both CT-based RENAL and PADUA scoring systems and HA3D nephrometry reconstruction. Associations between nephrometry scores and perioperative outcomes were assessed using Spearman’s correlation. Predictive performance for postoperative complications and early chronic kidney disease (CKD) was evaluated using receiver operating characteristic (ROC) analysis. **Results**: Overall, 41% and 35% of cases were downgraded according to three-dimensional (3D) RENAL and PADUA complexity–risk category assessment, respectively. Operative time demonstrated a moderate correlation with 3D RENAL (ρ = 0.57) and 3D PADUA (ρ = 0.49) scores. ROC curve analysis demonstrated numerical differences in area under the curve (AUC) values between 3D- and 2D-based nephrometry scores in predicting overall complications (RENAL: 0.61 vs. 0.54; PADUA: 0.69 vs. 0.46). 3D RENAL score demonstrated numerically higher AUC values for early postoperative CKD compared with 2D RENAL score (AUC: 0.72 vs. 0.67). **Conclusions**: HA3D-based nephrometry scores were associated with enhanced anatomical visualization, frequent downgrading of tumor complexity–risk categories, and numerical differences in predictive performance for postoperative complications and early renal functional decline compared with conventional CT-based scores. These findings suggest a potential role for HA3D modeling in preoperative planning for PN. However, given the limited sample size, these observations should be interpreted as exploratory and hypothesis-generating, and warrant validation in larger multicenter cohorts.

## Simple Summary

Accurate preoperative assessment of renal tumor anatomy is essential for planning partial nephrectomy and minimizing the risk of surgical complications while preserving kidney function. Conventional nephrometry scores are commonly derived from two-dimensional computed tomography images; however, these images may not fully represent the complex spatial relationships between the tumor and surrounding renal structures. Recently, advanced three-dimensional virtual models have been developed to provide a more detailed and intuitive visualization of kidney anatomy. In this prospective pilot study, we compared conventional imaging-based nephrometry scores with scores derived from hyperaccuracy three-dimensional models in patients undergoing minimally invasive partial nephrectomy. We aimed to explore whether three-dimensional assessment could better characterize tumor complexity and improve the prediction of postoperative complications and early renal functional decline. These findings may support future research on integrating three-dimensional modeling into preoperative planning and risk stratification for kidney tumor surgery.

## 1. Introduction

Over the past decade, technological advancements in imaging and intraoperative navigation have transformed surgical planning for renal tumors. These innovations are built on the principles of precision medicine and extended into the operative environment through the concept of precision surgery, which emphasizes tailoring interventions to each patient’s unique anatomical and functional characteristics [1]. In this context, 3D virtual reconstructions have gained significant traction for their ability to enhance anatomical visualization and improve preoperative planning [2]. Compared with traditional CT images, 3D reconstructions, particularly HA3D models, may provide a more intuitive and precise representation of tumor relationships with renal parenchyma, vasculature, and the collecting system, potentially improving preoperative assessment and reducing the cognitive burden of mentally reconstructing anatomy from 2D imaging [3,4]. Their integration into surgical workflows has shown potential benefits, including optimized clamping strategies, improved tumor localization, and more confident dissection during nephron-sparing procedures [5].

Amid this rapidly evolving landscape, nephron-sparing surgery (NSS) provides an ideal clinical setting to leverage precision surgery principles. PN is the standard treatment for T1 renal tumors, offering oncological outcomes comparable to those of radical nephrectomy while preserving greater renal function [6,7,8]. Increasing evidence suggests that PN can also be safely performed in carefully selected elderly patients, providing favorable surgical, functional, and oncologic outcomes when compared with radical nephrectomy [9]. Although alternative nephron-sparing approaches, such as percutaneous thermal ablation, are increasingly used in selected patients, relevant complications, including tumor tract seeding, have been reported, underscoring the importance of careful patient selection [10]. However, the technical complexity of the procedure can vary significantly depending on the tumor’s anatomical characteristics, thereby directly influencing the risk of intra- and postoperative complications [11]. Advances in surgical techniques, including selective and zero-ischemia strategies, have further emphasized the importance of accurate preoperative anatomical assessment when planning nephron-sparing surgery [12,13]. To support surgical planning and risk stratification, several nephrometry scores have been developed, the most widely used being RENAL and PADUA [14,15]. Additional systems, such as the C-index and the arterial-based complexity score, have also been proposed to better quantify tumor location and vascular complexity [16,17,18]. These scoring systems are based on parameters derived from conventional axial CT images and are used to assess the complexity of the renal tumor and to predict potential clinical outcomes, including operative times, ischemia, and the risk of complications [19]. The introduction of HA3D models has opened up new perspectives in preoperative planning [3]. Despite these advantages, evidence supporting the reliability of 3D-based modeling for predicting perioperative outcomes remains limited, even as it is increasingly adopted in PN, underscoring the need for prospective comparisons with traditional CT-derived nephrometry systems [4]. However, prospective studies directly comparing conventional CT-based nephrometry with HA3D-derived nephrometry scores in predicting perioperative outcomes remain limited.

The aim of this prospective pilot study is to compare RENAL and PADUA scores calculated from conventional CT imaging with HA3D-derived nephrometry scores in patients undergoing robotic or laparoscopic PN. The primary aim is to evaluate how each imaging approach predicts postoperative complications, their concordance, and the potential for downgrading score or complexity–risk category using 3D models.

## 2. Materials and Methods

### 2.1. Study Design and Participants

We prospectively enrolled 17 consecutive patients with intermediate- or high-complexity (RENAL ≥ 7) and moderate- or high-risk (PADUA ≥ 8) renal tumors from April 2025 to November 2025 at our public tertiary referral center. Patients with a contrast-enhanced CT scan finding of an organ-confined (T1–T2) renal mass and suitable for PN were enrolled. All consecutive eligible patients during the study period were enrolled to minimize potential selection bias.

### 2.2. Nephrometry Scores Assessment

#### 2.2.1. Conventional CT-Based Nephrometry Score Assessment

All patients underwent a preoperative multiphase contrast-enhanced CT scan. Nephrometry evaluation was performed on standard CT axial, coronal, and sagittal plane images. Tumor complexity was assessed using established nephrometry systems, RENAL and PADUA scores, based on tumor size, exophytic/endophytic rate, longitudinal and rim location, proximity to the collecting system and renal sinus, and relationships with renal vessels. All scores were calculated by a single experienced urologist familiar with both nephrometry systems to ensure consistent application of the scoring criteria across cases. Scoring was performed based exclusively on imaging findings and was not influenced by postoperative outcomes. Tumors were categorized as intermediate- or high-complexity or moderate- or high-risk according to validated cut-offs.

#### 2.2.2. HA3D Model Generation

HA3D virtual models were generated from the same preoperative CT datasets. DICOM images were processed by bioengineers from Medics3D Srl (Turin, Italy), using dedicated segmentation software to reconstruct patient-specific 3D models of the kidney, including renal parenchyma, tumor, arterial and venous branches, renal sinus and collecting system. The resulting models were reviewed for anatomical accuracy and made available for preoperative planning.

#### 2.2.3. HA3D-Based Nephrometry Assessment

**Figure 1:**
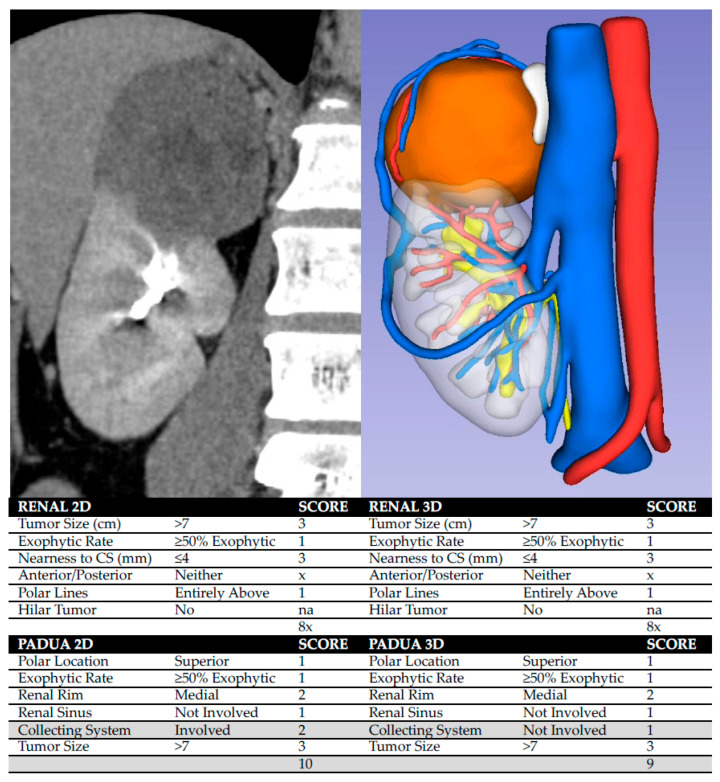
Comparison between a 2D CT urographic coronal image and an HA3D model. (**Left panel**): Conventional 2D CT imaging used for RENAL and PADUA score calculation, in which the collecting system (CS) appears to be involved. (**Right panel**): HA3D model showing a volumetric representation of the renal tumor, renal parenchyma, arterial and venous anatomy, and a non-involved collecting system. In this case, HA3D-based assessment allowed improved visualization of the CS, influencing PADUA score assignment and resulting in downgrading from a high- to a moderate-risk category.

Nephrometry evaluation was repeated using the HA3D models. Tumor anatomical features corresponding to RENAL and PADUA score components were reassessed in a 3D environment, allowing direct visualization of spatial relationships between the tumor and critical renal structures. HA3D-derived scores were calculated by the same experienced urologist to ensure consistent application of the criteria, and compared with conventional CT-based scores. Any change in overall score or complexity–risk category was recorded. Figure 1 illustrates how HA3D model visualization may refine anatomical assessment compared with 2D CT images, potentially explaining the observed downgrading of complexity–risk categories.

### 2.3. Surgical Technique and Approach

Partial nephrectomies were performed with the assistance of the DaVinci Xi robot (Intuitive Surgical, Inc., Sunnyvale, CA, USA) or laparoscopically by three experienced surgeons (A.C., G.Z., and T.S.), depending on the availability of the robotic platform. Transperitoneal or retroperitoneal approaches were used depending on tumor location and patient abdominal characteristics (e.g., posterior tumors and/or previous abdominal surgery), as well as the surgeons’ preference.

### 2.4. Data Collection

For each patient enrolled, demographic and preoperative clinical parameters were prospectively collected, including: age, gender, body mass index (BMI), age-adjusted Charlson Comorbidity index (CCI) [20], American Society of Anesthesiologists (ASA) score [21], and baseline laboratory tests such as hemoglobin (HB), serum creatinine (sCr) and estimated glomerular filtration rate (eGFR). Preoperative radiological data included tumor clinical T stage, size, location, side, number of arteries, number of veins, number of accessory arteries, and surgical complexity–risk, as evaluated via CT and 3D models, according to RENAL and PADUA scores. Intraoperative and anesthesiological data included surgical technique (robot-assisted or laparoscopic), approach (transperitoneal or retroperitoneal), operative duration, management of renal artery, warm ischemia time (WIT), estimated blood loss (EBL), anesthesia duration, intraoperative (IO) fluid administration, and IO diuresis. Postoperative outcome data included HB, sCr, eGFR on days 0, 1, 2, and 90 days, complications according to the Clavien–Dindo classification [22], and hospital stay. Pathological data included pathological stage, histological type, grading system according to the WHO and the International Society of Urological Pathology (ISUP) [23], and the presence of positive margins.

### 2.5. Statistical Analysis

Descriptive statistics were presented as medians and interquartile ranges (IQR) for continuous variables and as counts and percentages for categorical variables. Spearman’s rank correlation coefficient (ρ) was used to assess the relationship between 2D-based and 3D-based nephrometry scores and perioperative outcomes, including EBL, operative time, early postoperative CKD, and overall postoperative complications. Functional outcome was evaluated using the incidence of de novo early CKD, defined as a decrease in eGFR to <60 mL/min/1.73 m^2^ at 90 days post-surgery in patients with a preoperative eGFR ≥ 60 mL/min/1.73 m^2^. Correlation matrices were visualized as heat maps. Confidence intervals (CIs) for Spearman correlation coefficients were estimated using bootstrap resampling. Both benign and malignant tumors were included in the analyses, as perioperative complications and renal functional outcomes are primarily related to surgical complexity rather than tumor histology. ROC curve analysis was performed to explore the discriminative ability of CT-based and HA3D-based nephrometry scores for predicting postoperative complications and early renal functional decline. Discrimination was quantified using the AUC with its corresponding 95% CI, calculated via bootstrap resampling. Given the limited cohort size and number of outcome events, ROC analyses were intended as exploratory assessments of potential trends rather than definitive estimates of predictive performance. All statistical analyses were conducted using R software (version 4.5.3; R Foundation for Statistical Computing, Vienna, Austria). ROC curves and AUCs were obtained with the pROC package (version 1.19.0.1), and heat maps were generated using the pheatmap package (version 1.0.13).

## 3. Results

A total of 17 patients were enrolled in this study. Overall, 12 were male (71%), and five were female (29%), with a median age of 65 years (IQR 55–74). Overall, 12 tumors were staged as cT1a (71%), four as cT1b (24%), and one as cT2a (5.9%). Demographic and preoperative clinical data are reported in Table 1. The median hospital stay was 6 days (IQR 6–7). Among the 17 lesions, eight tumors were ccRCC (47%), four were papillary RCC (24%), three were oncocytoma (18%), one was angiomyolipoma (5.9%), and one was chromophobe RCC (5.9%). Both benign and malignant lesions were included in the perioperative outcome analyses. None had a positive margin. Intraoperative and postoperative data are reported in Table 2. Postoperative day 2 laboratory values showed a median HB of 117 g/L (IQR 112–123), a median sCr of 1.13 mg/dL (IQR 0.94–1.27), and a median eGFR of 68 mL/min/1.73 m^2^ (IQR 54–78). Postoperative complications occurred in 7 patients (41%). According to the Clavien–Dindo classification, one patient (5.9%) experienced a grade I complication, five patients (29%) had grade II complications, and one patient (5.9%) had a grade III complication. Overall, six complications (35%) were classified as minor (Clavien–Dindo I–II), while one (5.9%) was classified as major (Clavien–Dindo ≥ III). The median time to complication was 2 days (IQR 2–5). HB data at 90 days were available for 14 patients (82%), with a median HB level of 135 g/L (IQR 125–144). sCr and eGFR data at 90 days were available for all patients, with a median value of 1.07 mg/dL (IQR 0.94–1.14) and 74 mL/min/1.73 m^2^ (IQR 60–80), respectively. According to 90-day eGFR values, four patients (24%) met the predefined criterion for de novo early postoperative CKD.

**Figure 2:**
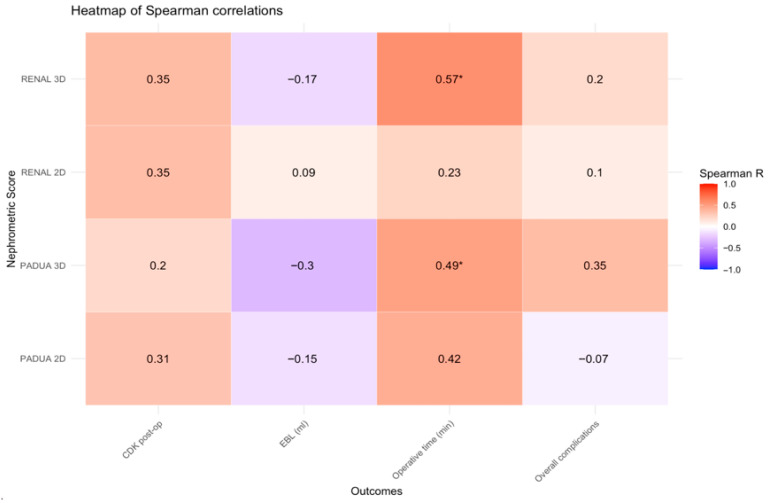
Heat map showing Spearman’s correlations between 3D-based and 2D-based nephrometry scores and postoperative outcomes, including early CKD, EBL (mL), operative time (min), and overall complications. Asterisks (*) indicate statistical significance. The bar on the right side of the map indicates the color legend of the Spearman correlation coefficients.

**Figure 3:**
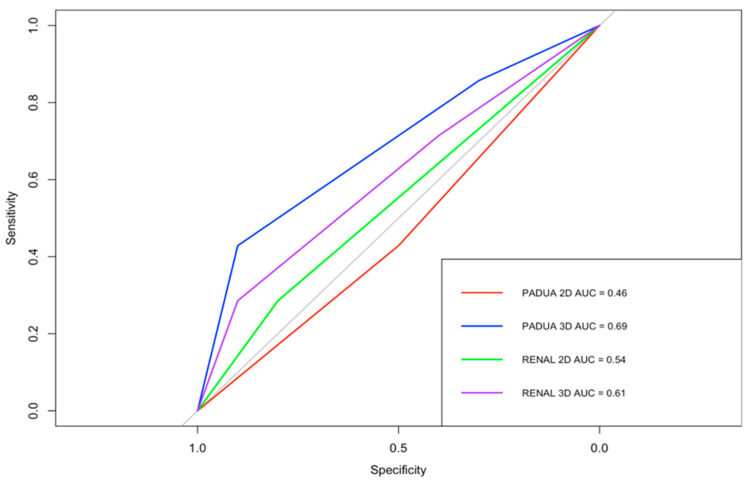
ROC curves demonstrating the discriminative ability of 2D- and 3D-based RENAL and PADUA nephrometry scores for predicting overall postoperative complications. ROC curves represent exploratory observations. AUC values are presented with 95% confidence intervals derived from bootstrap resampling (RENAL: 3D 95% CI 0.33–0.86 vs. 2D 95% CI 0.33–0.76; PADUA: 3D 95% CI 0.42–0.91 vs. 2D 95% CI 0.22–0.71). Statistical comparison using the DeLong test showed no significant differences in predictive performance between 2D and 3D models (*p* > 0.05).

**Figure 4:**
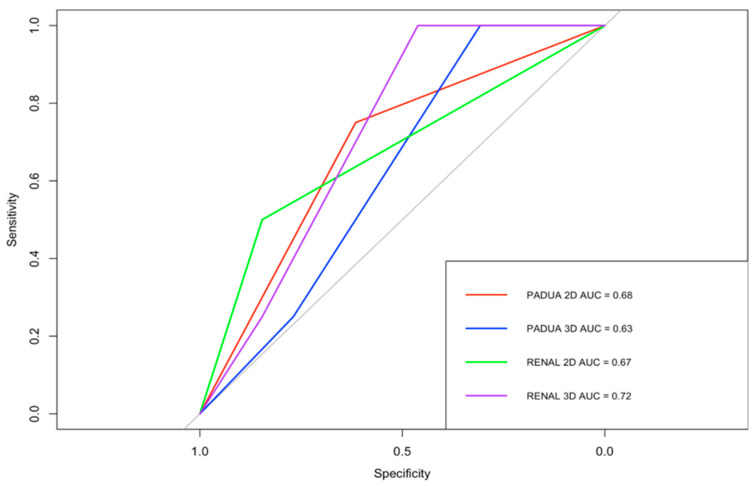
ROC curves demonstrating the discriminative ability of 2D- and 3D-based RENAL and PADUA nephrometry scores for predicting early postoperative CKD. ROC curves represent exploratory observations. AUC values are presented with 95% confidence intervals derived from bootstrap resampling (RENAL: 3D 95% CI 0.50–0.93 vs. 95% CI 0.39–0.96; PADUA: 3D 95% CI 0.38–0.87 vs. 2D 95% CI 0.37–0.91). Given the limited number of CKD events, formal statistical comparison between ROC curves was not performed.

According to 2D RENAL nephrometry score, 13 lesions (77%) were classified as intermediate complexity, and four (23%) as high complexity. According to 3D-based RENAL assessment, eight lesions (47%) demonstrated a change in overall score, while nine (53%) remained unchanged. Overall, seven lesions (41%) changed complexity category: six were downgraded from intermediate to low, and one was downgraded from high to moderate. According to 2D PADUA nephrometry score, nine lesions (53%) were classified as moderate risk and eight (47%) as high risk. According to 3D-based PADUA, eight lesions (47%) demonstrated a change in overall score, while nine (53%) remained unchanged. Overall, six lesions (35%) changed risk category: four were downgraded from high to moderate, and two from intermediate to low risk, as shown in Table 3. Using Spearman’s rank correlation analysis, operative time demonstrated moderate positive correlations with the 3D-based RENAL and PADUA scores (ρ = 0.57, 95% CI: 0.11–0.85 and ρ = 0.49, 95% CI: 0.06–0.80, respectively). In contrast, weaker correlations were observed for the corresponding 2D-based scores (Figure 2). Figure 3 illustrates the performance of 2D-based and 3D-based nephrometry scores in predicting overall postoperative complications. According to ROC analysis, the AUC values differed numerically between 3D- and 2D-based assessments (RENAL: 0.61, 95% CI 0.33–0.86 vs. 0.54, 95% CI 0.33–0.76; PADUA: 0.69, 95% CI 0.42–0.91 vs. 0.46, 95% CI 0.22–0.71). However, formal statistical comparison of the ROC curves using the DeLong test did not demonstrate statistically significant differences between the models (all *p* > 0.05). A similar exploratory analysis was performed for early postoperative CKD. Figure 4 shows the ROC curves of 2D- and 3D-based nephrometry scores for predicting early postoperative CKD. 3D-based RENAL assessments showed numerically higher AUC values than 2D-based assessments (0.72, 95% CI 0.50–0.93 vs. 0.67, 95% CI 0.39–0.96), whereas PADUA demonstrated the opposite trend (2D: 0.68, 95% CI 0.37–0.91 vs. 3D: 0.62, 95% CI 0.38–0.87). Given the limited number of CKD events, formal statistical comparison between ROC curves was not performed.

## 4. Discussion

Since their introduction, nephrometry scoring systems have provided an objective method for quantifying renal tumor complexity, supporting surgical planning for PN. Early classifications, including RENAL and PADUA systems, were designed to standardize preoperative risk assessment using anatomical variables derived from 2D contrast-enhanced CT imaging. These tools facilitated a more structured description of tumor size, depth, and spatial relationships with renal structures, and are widely adopted to predict operative difficulty and postoperative complications [14,15]. However, traditional nephrometry scores have demonstrated a modest ability to predict perioperative outcomes across heterogeneous patient populations. Their performance is limited by the inherent constraints of 2D imaging, which captures anatomy in axial, coronal, and sagittal planes but fails to fully convey the 3D relationships between the tumor, renal vasculature, and collecting system [24]. As observed in prior studies, urologists must mentally reconstruct spatial anatomy from serial CT slices. This cognitively demanding process introduces observer variability and may lead to inaccurate estimates of complexity [24,25].

These limitations provided the rationale for developing 3D virtual reconstructions and 3D-based nephrometry systems. By processing standard CT datasets into interactive volumetric models, HA3D techniques enable direct visualization of tumor morphology, vascular anatomy, and relationships between renal sinus and collecting system [26]. Early clinical investigations demonstrated that 3D virtual models not only improve anatomical understanding but also frequently lead to downgrading of tumor complexity–risk categories compared with 2D CT-based scoring, suggesting that 3D visualization may lead to different assessments of tumor complexity compared with conventional 2D imaging [3].

In the present study, we applied this concept by directly comparing conventional CT-based nephrometry with HA3D-derived scores to evaluate their ability to predict postoperative complications and renal functional outcomes in a minimally invasive PN setting. In this study, 3D-based nephrometry assessment resulted in downgrading 41% and 35% of cases according to RENAL complexity and PADUA risk categories, respectively. Downgrading may reflect the enhanced anatomical visualization provided by HA3D models, which allows a clearer depiction of tumor depth, endophytic components, and proximity to critical structures, such as renal sinus or urinary collecting system, which may facilitate a more precise assessment of technical difficulty. These findings are consistent with previous work demonstrating that 3D virtual models improve the assessment of surgical complexity. Porpiglia et al. reported that 3D virtual reconstructions led to downgrading of RENAL and PADUA scores in approximately 52.4% and 48.5% of cases, respectively [3].

Moreover, these observations may suggest that 3D-based nephrometry is more strongly associated with intraoperative parameters. In particular, 3D-based RENAL and PADUA scores demonstrated moderate correlations with operative time (ρ = 0.57 and ρ = 0.49, respectively). Operative time is a surrogate measure of surgical difficulty, and these findings are consistent with the hypothesis that HA3D assessment better captures anatomical features associated with surgical difficulty encountered during NSS.

The predictive performance for postoperative complications may provide preliminary indications of the potential utility of HA3D scoring. ROC analysis showed numerically different AUC values between HA3D-based and 2D-based scores in predicting overall complications (RENAL: 0.61 vs. 0.54; PADUA: 0.69 vs. 0.46). The low predictive performance of the conventional 2D scores in this series likely reflects a restricted case-mix and the selection bias inherent to a small cohort of exclusively intermediate- to high-complexity tumors, rather than a failure of the scoring systems themselves. Although modest, these observations may suggest that enhanced anatomical visualization could contribute to improved risk stratification. However, given the limited cohort size and overlapping CIs, these differences should be interpreted as exploratory trends rather than statistically demonstrated improvements. Similar findings have been reported in previous studies evaluating 3D-derived nephrometry systems in robotic-assisted partial nephrectomy (RAPN). Similarly, Bianchi et al. demonstrated that 3D-derived RENAL and PADUA scores performed with higher accuracy in predicting complications after RAPN than conventional CT-based scores, highlighting the enhanced anatomical clarity provided by 3D models [27]. More recently, Amparore et al. confirmed these findings in a multi-institutional cohort of 318 patients, showing that 3D models improved interrater concordance and outperformed 2D imaging in predicting both overall and major complications [25]. These findings are consistent with those reported by Huang et al., who demonstrated that integrating 3D visualization into SPARE scoring significantly improved predictive performance for Tetrafecta outcomes [28]. Collectively, these studies provide converging evidence that 3D nephrometry scores represent a meaningful evolution in preoperative assessment for PN.

Beyond agreement with prior work, our results exploratorily suggest a potential relationship with early postoperative CKD prediction. While previous studies evaluated complication endpoints, we observed that the HA3D-based RENAL score showed a numerically higher AUC for predicting early CKD at 90 days (AUC 0.72 vs. 0.67 for 3D vs. 2D), whereas PADUA demonstrated the opposite trend. This divergence may reflect differences in scoring architecture: RENAL score incorporates radius and endophytic depth, features that directly influence nephron preservation, whereas PADUA score assigns greater weight to collecting system involvement, which may influence complications more than renal function. This hypothesis aligns with the growing recognition that tumor contact surface area, and parenchymal volume excised correlate with functional decline independent of WIT.

Mechanistically, the improved performance of HA3D-based scoring likely arises from superior spatial depiction of tumor–kidney relationships. HA3D models allow visualization of features that cannot be fully appreciated on axial CT alone [24]. In contrast, HA3D reconstructions reduce abstraction by providing direct volumetric visualization, enabling more accurate evaluation of endophytic components, sinus proximity, and urinary collecting system [3,26]. The widespread adoption of robotic platforms has further expanded the feasibility of nephron-sparing surgery for anatomically complex renal tumors and has facilitated the integration of advanced imaging technologies into surgical planning and intraoperative workflows [29]. Our findings may support these interpretations, as complexity downgrading using HA3D resulted in fewer lesions in high-risk categories, with possible implications for surgical planning and patient counseling.

From a clinical standpoint, the observed differences in predictive performance may have implications for precision surgery. A more refined preoperative anatomical assessment may help guide decisions regarding selective arterial clamping, which has been associated with improved postoperative renal function in previous studies [3]. In this context, intraoperative changes from off-clamp to on-clamp techniques during robotic partial nephrectomy have been reported, highlighting the importance of accurate preoperative prediction of surgical complexity and vascular anatomy [30]. Indeed, the 3D literature consistently reports increased adoption of selective and super-selective clamping strategies, reduced collecting system injuries, and more confident dissection during RAPN [26,27]. Improved preoperative understanding of vascular anatomy may reduce intraoperative uncertainty, decrease operative time, and minimize ischemia without compromising oncological safety. Furthermore, precise risk stratification may optimize perioperative resource allocation, guide patient selection, and support shared decision-making in cases where radical nephrectomy is being considered for borderline tumors. Within the framework of precision medicine, HA3D-based nephrometry may serve as a complementary tool for patient-specific surgical planning rather than population-based risk estimation.

Nevertheless, several limitations warrant consideration. The small sample size limits statistical power and may lead to unstable AUC estimates. Accordingly, ROC analyses should be interpreted cautiously and considered primarily exploratory. The single-center design reduces generalizability and may reflect institution-specific expertise in RAPN and 3D planning. Nephrometry scores were assessed by a single experienced urologist; therefore, interobserver variability and inter-rater concordance could not be evaluated. Future studies including multiple independent raters are warranted to assess the reproducibility of HA3D-based nephrometry assessment. The availability and cost of HA3D modeling remain barriers to widespread adoption, and the time required for modeling may limit intraoperative integration. The 90-day evaluation reflects early renal functional decline, and longer follow-up with longitudinal functional assessment is needed to confirm persistent CKD. Given the limited sample size and event counts, these findings should be interpreted cautiously and considered primarily hypothesis-generating observations requiring validation in larger prospective cohorts.

Despite these limitations, this study has several strengths. First, the prospective design allowed standardized data collection; however, these observations remain exploratory given the limited sample size. Second, all cases were scored using both 2D CT and HA3D models derived from the same imaging dataset, enabling direct within-patient comparisons and reducing anatomical heterogeneity as a confounding factor. Third, the study focused on intermediate- and high-complexity renal masses, representing a clinically relevant population in which anatomical assessment and surgical planning are most challenging. Finally, postoperative outcomes included both complications and renal functional parameters at multiple timepoints, strengthening the clinical relevance of the findings beyond pure feasibility endpoints.

Future work should explore the cost-effectiveness of integrating 3D modeling into clinical workflows, particularly as semi-automated and AI-based segmentation platforms become increasingly available. Standardization of 3D-derived nephrometry scoring criteria is needed to reduce heterogeneity across institutions and studies. Longer-term follow-up should evaluate whether improved perioperative prediction translates into better preservation of renal function and reduced progression to CKD. Integration with intraoperative navigation platforms, augmented reality overlays, and robotic guidance systems represent another promising direction that aligns with ongoing advances in precision surgery. Finally, as 3D imaging improves lesion characterization, research should investigate whether nephrometry can support treatment decision-making between active surveillance, ablation, partial nephrectomy, and radical nephrectomy.

## 5. Conclusions

In conclusion, this pilot study demonstrates the feasibility of HA3D-based nephrometry for assessing renal tumor complexity. While our preliminary data suggest a potential for improved risk stratification, the small cohort and observed instability in predictive estimates preclude definitive claims of superiority over conventional 2D CT-based scoring. Larger, multicenter studies are needed to validate these findings and determine whether integrating HA3D nephrometry into surgical planning can meaningfully improve patient outcomes in the era of precision urologic oncology.

## Figures and Tables

**Table 1:** Demographic and preoperative clinical data.

Median BMI kg/m^2^ (IQR)	24.3 (22.1–30.7)
**Age-adjusted CCI, points (%)**	
0	2 (12)
1	1 (5.9)
>2	14 (82)
**ASA score, n (%)**	
1	1 (5.9)
2	12 (71)
3	4 (24)
Median HB g/L (IQR)	144 (138–148)
Median sCr mg/dL (IQR)	0.93 (0.84–1.13)
Median eGFR mL/min/1.73 m^2^ (IQR)	85 (63–90)
Median tumor size, mm (IQR)	37 (29–45)
**Tumor location, n (%)**	
Middle third	14 (82)
Inferior third	2 (12)
Upper third	1 (5.9)
**Tumor side, n (%)**	
Right	10 (59)
Left	7 (41)
**Accessory artery n (%)**	
1	1 (5.9)
2	1 (5.9)

Abbreviations: ASA, American Society of Anesthesiologists; BMI, Body Mass Index; CCI, Charlson Comorbidity Index; eGFR, estimated Glomerular Filtration Rate; HB, Hemoglobin; sCr, serum Creatinine.

**Table 2:** Intraoperative and postoperative data.

Surgical Technique and Approach, n (%)	
Transperitoneal RAPN	8 (47)
Retroperitoneal RAPN	5 (29)
Laparoscopic transperitoneal PN	2 (12)
Laparoscopic retroperitoneal PN	2 (12)
Median operating duration, minutes (IQR)	174 (155–180)
**Renal hilum management, n (%)**	
On-clamp	15 (88)
Off-clamp	2 (12)
Median WIT duration, minutes (IQR)	23 (19–32)
Median EBL, mL (IQR)	100 (50–100)
Median anesthesia duration, minutes (IQR)	270 (240–285)
Median IO fluid administration, mL (IQR)	1500 (1500–1700)
Median IO diuresis, mL (IQR)	300 (200–500)
**Histology, n (%)**	
Malignant	13 (77)
Benign	4 (23)
**Pathological T, n (%)**	
pT1a	10 (59)
pT1b	3 (18)
na	4 (24)
**ISUP grade, n (%)**	
G1	3 (18)
G2	5 (29)
G3	4 (24)
na	5 (29)

Abbreviations: EBL, Estimated Blood Loss; IO, Intraoperative; ISUP, International Society of Urological Pathology; na, not applicable; PN, Partial Nephrectomy; RAPN, Robotic-Assisted Partial Nephrectomy; WIT, Warm Ischemia Time.

**Table 3:** Contingency tables showing the distribution of tumor complexity/risk categories according to 2D- based and 3D-based RENAL and PADUA scores.

RENAL	Low	Intermediate	High	Total 2D
Low	0	0	0	0
Intermediate	6	7	0	13
High	0	1	3	4
**Total 3D**	6	8	3	17
**PADUA**	**Low**	**Moderate**	**High**	**Total 2D**
Low	0	0	0	0
Moderate	4	5	0	9
High	0	4	4	8
**Total 3D**	4	9	4	17

## Data Availability

The datasets generated during and/or analyzed during the current study are available from the corresponding author on reasonable request.

## Abbreviations

The following abbreviations are used in this manuscript:

2D	Two-Dimensional
3D	Three-Dimensional
ASA	American Society of Anesthesiologists
AUC	Area Under the Curve
BMI	Body Mass Index
CCI	Charlson Comorbidity Index
ccRCC	clear cell Renal Cell Carcinoma
CIs	Confidence Intervals
CKD	Chronic Kidney Disease
CS	Collecting System
CT	Computed Tomography
EBL	Estimated Blood Loss
eGFR	estimated Glomerular Filtration Rate
HA3D	Hyperaccuracy Three-Dimensional
HB	Hemoglobin
IO	Intraoperative
IQR	Interquartile Ranges
ISUP	International Society of Urological Pathology
NSS	Nephron-Sparing Surgery
PN	Partial Nephrectomy
RAPN	Robotic-Assisted Partial Nephrectomy
ROC	Receiver Operating Characteristic
sCr	serum Creatinine
WIT	Warm Ischemia Time
